# The American Association for the Advancement of Science

**Published:** 1853-08

**Authors:** 


*The American Association for the Advancement of Science.*

THE seventh regular session of this body was held, commencing July 28th, in Cleveland. A large number of scientific men from different parts of our country were present and participated in the exercises. The following papers were read during the meeting:

On the value of the Barometer in navigating the American Lakes. By W. C. Redfield, of New York.

Investigations in Analytical Morphology, No. 4; Stability of Saturn's Ring. By Prof. B. Pierce, of Cambridge, Mass.

Notice of a Hail Storm which passed over New York, July 1, 1853. By Prof. E. Loomis, of New York.

Recent Discovery of a Deep-sea Bank on the eastern side of the Gulf Stream, off the coast of South Carolina, Georgia and Florida, by Lieuts. Commanding Craven and Maffit, U. S. N., Ass'ts, Coast Survey. Presented by Prof. Bache, Supt.

On the Resistance of the Vertical Plates of Tubular Bridges. By Herman Haupt, Supt. of the Pennsylvania Central Rail Road, Phila.

On the Tides at Key West, Florida, from observations made in connection with the U. S. Coast Survey. By Prof. A. D. Bache, Supt. Washington.

The Zodiacal Light, the periodical appearance of Meteors, and the point in space to which the motion of the Solar System is directed. By Daniel Vaughan, of Cincinnati.

On the South East Monsoon of Texas, the Northers of Texas and the Gulf of Mexico, and the Abnormal Atmospheric Movements of the North American Continent generally. By Lorin Blodgett, of Washington.

Investigations in Analytical Morphology, No. 2; Stable and Unstable forms of Equilibrium. By Prof. B. Pierce, of Cambridge, Mass.

An Investigation of the Storm Curve, deduced from the Relations existing between the direction of the Wind, and the Rise and Fall of the Barometer. By Prof. J. H. Coffin, of Easton, Pa.

Does the Moon exert a sensible influence upon the Clouds? By Prof. E. Loomis, of New York.

On the Rising of Water in Springs immediately before Rain. By Prof. John Brocklesby, of Trinity College, Hartford.

A Geological Reconnoisance of the Arkansas River. By Dr. J. A. Warder, of Cincinnati.

On the Blood Corpuscle—holding Cells, and their relation to the Spleen. By Dr. W. I. Burnett, of Boston.

Origin of Quartz Pebbles in the Sandstone Conglomerate, and the Formation of the Silicious Stratified Rocks. By Prof. J. Brainard, of Cleveland.

Indications of Weather, as shown by Animals and Plants. By W. H. B. Thomas, of Cincinnati.

On the Geology of the Choctaw Bluff. By A. Winchell, Eutaw, Alabama.

On six Species of Plants. By Prof. Alphonso Wood, of Cin'ti.

On a remarkable Class of conjunct bases containing Cobalt and the Elements of Ammonia. By Dr. F. A. Genth, and Dr. W. Gibbs.

On the Solidification of Coral Reefs of Florida, and Source of Carbonate of Lime in the growth of Corals. By Prof. E. N. Horsford, of Cambridge.

On the fatal effects of Chloroform. By Prof. E. N. Horsford, of Cambridge, Mass.

Investigation of the power of the Greek Z, by means of Phonetic Laws. By Prof. S. S. Haldeman, of Columbia, Pa.

Notes on the Specimens of the bottom of the Ocean, brought up in recent explorations of the Gulf Stream, in connection with the Coast Survey. By L. F. Pourtales, Ass't. Presented by Prof. Bache, Supt.

On the Earthquake of April 29. 1852. By Lorin Blodgett, of Washington.

Investigations in Analytical Morphology, No. 3; Forms of the Elastic Sack. By Prof. B. Pierce, of Cambridge, Mass.

On some special Analogies of Structure in the Eastern Hemisphere of the Earth and the visible Hemisphere of the Moon, with conjectures as to the Structure and Appearance of those Portions of the Moon which are invisible. By Prof. Stephen Alexander, of Princeton.

On the Measurement of the Heights by Barometer. By Prof. E. Loomis, of New York.

On the Tides of the Western Coast of the United States, from observations at San Fransisco, California, in connection with the U. S. Coast Survey. By Prof. A. D. Bache, Supt., Wash'n.

On the distribution of percipitation in Rain and Snow on the North American Continent. By Lorin Blodgett, of Wash'n.

Remarks on Lithography and lithographic Transfers. By Lt. E. B. Hunt.

On the Method of finding the error of a Chronometer by equal altitudes of the Sun. By Prof. W. Chauvenet, of the U. S. Naval Academy.

On the distribution of Heat over the North American Continent, and the construction of its Isothermal lines. By Lorin Blodgett, of Washington.

On the Formation and mode of Development of the Renal Organs in Vertebrata. By Dr. W. I. Burnett, of Boston.

On the Formation and Functions of the Allantois. By Dr. W. I Burnett, of Boston.

Researches on the Development of the Viviparous Aphides. By Dr. W. I. Burnett, of Boston.

On the parallelism of the lower Silurian groups of Middle Tennessee with those of New York. By Prof. J. M. Safford, of Lebanon, Tenn.

On the Binocular Microscope. By Prof. J. L. Riddell, N. Orleans.

On the Histology of Red Blood. By Prof. J. L. Riddell, of New Orleans.

On the origin of Capillary Blood-Vessels. By Prof. J. L. Riddell, of New Orleans.

Investigations in Analytical Morphology, No. 1, Description of the Science. By Prof. B. Pierce, of Cambridge, Mass.

On some Relations of the Central Distances of the primary Planets, Satellites, and Rings of the Solar System of which Bode's Law would seem to be but an imperfect expression. By Prof. Stephen Alexander, of Princeton.

On Personal Equations in Astronomical observation. By Dr. B. A. Gould, Jr., of Cambridge, Mass.

A new method of securing uniform circular motion in the machinery used in receiving the registration of Astronomical observations of right ascension. By Prof. O. M. Mitchel, Cincinnati Obs.

Personal Scale of Astronomical Observers. By Prof. B. Pierce, of Cambridge, Mass.

On the comparative precision of the Electro-chronographic, or

American Method of Observation. By Dr. B. A. Gould, Jr., of Cambridge, Mass.

A singular case of internal fringes, produced by interference in the eye itself. By Prof. Joseph Lovering, Cambridge, Mass.

Account of Experiments on Sound. By Capt. Wilkes.

Mathematical Analysis of the Contact of Surface in Oscillating machinery. By Prof. C. W. Hackley, of New York.

On Cohesion of Fluids, Evaparation, and Steam Boiler Explosions. By Lt. E. B. Hunt.

Project of a Geographical Department of the Library of Congress. By Lt. E. B. Hunt.

On the Structure and Transformation of Oscillaria Aureliana. By Prof. J. L. Riddell, of New Orleans.

On the Effect of the Reclamation of the annually Inundated Lands of the Mississippi Valley upon the general Health of the country, and the navigation of that River. By Andrew Brown, of Natchez, Miss.

On the Structure and Affinities of certain Fossil plants of the carboniferous era. By J. S. Newberry, M.D., of Cleveland.

Notice of Bradford's Machine for separating Metals by their specific gravity. By Capt. Wilkes.

On some points in the History of Gordius. By S. N. Sanford, of Granville, Ohio,

On the Geological Age and Affinities of the Fossil Fishes which belong to the sandstone formations of Connecticut, New Jersey, and the coal field near Richmond, in Virginia. By W. C. Redfild, of New York.

On the Wheat Fly and its ravages. By R. Howell, of Nichols, New York.

The Conical Condenser, a telescopic Appendage. By Lt. E. B. Hunt.

On the primitive Form and Dimensions of the Asteroid Planet, the cause of the Instability of the same, and of the Varieties in the Orbits of the Asteroids. By Prof. Stephen Alexander, of Princeton.

Criterion for the Rejection of Doubtful Observations. By Prof. B. Pierce; of Cambridge, Mass.

Some errors peculiar to the observer, which may affect determinations of the declinations of the fixed Stars. By Prof. John H. Coffin, of the Washington Observatory.

New Formulas of spherical Trigonometry. By Prof. W. Chauvenet, of the U. S. Naval Academy.

Theory of the Action of Neptune upon Saturn. By Prof. B. Pierce, of Cambridge, Mass.

On the Limit towards which the series;

$$1 + \frac{1}{2^{1+p}} + \frac{1}{3^{1+p}} + \frac{1}{4^{1+p}} + \&c.$$

Converges for p=0. By Dr. Julius Friedlander, of Berlin.

On the Subordination of Atmospheric Phenomena, or the Position of the several Classes with respect to the primary Cause or initiatory Processes. By Lorin Blodgett, of Washington.

Strictures on the Mechanical explanation of the zigzag path of the Electric Spark. By Prof. O. N. Stoddard, of Miami University.

Theory of Molecular Forces. Explanatory of the gaseous, liquid, and solid conditions of matter. By Prof. J. L. Riddell, of New Orleans.

On the Winds of the coast of the United States on the Gulf of Mexico. By Prof. A. D. Bache, Supt. U. S. Coast Survey, Washington.

On the Velocity of transmission of Electric Signals along iron Telegraph wires. By Dr. B. A. Gould, Jr., of Cambridge, Mass.

Illustrations of Cohesion. By Prof. Joseph Henry, of Washington.

On a Modification of Soleil's polarising apparatus for projection. By Prof. Joseph Lovering, Cambridge, Mass.

On the Barometric Pressure in extreme Latitudes, and the existence of Belts of low Barometer in the Arctic Regions. By L. Blodgett, of Washington.

On optical Meteorology. By Professor Joseph Lovering, Cambridge, Mass.

On the Carboniferous Flora in Ohio with Descriptions of fifty new Species of Fossil Plants. By Dr. J. S. Newberry, of Cleveland.

The Artesian Well, Charleston. By the Rev. P. R. Lynch, of Charleston.

Account of the recent American Scientific Expeditions. By Prof. S. F. Baird, Washington.

On the Fossil Fishes of the Cliff Limestone of Ohio. By Dr. J. S. Newberry, of Cleveland.

On the Reproduction of the Toad and Frog, without the intermediate stage of Tadpole. By Dr. W. I. Burnett, of Boston.

On the Signification of Cell Segmentation. By Dr. W. I. Burnett, of Boston.

Description of a Plan for furnishing a fluid Mirror, to be used in a Reflecting Telescope. By Prof. George R. Perkins.

Several of these papers, especially those of Drs. Riddell and Burnett and Prof. Horseford, are of interest to the medical profession. We have only room in this number for an abstract from Prof. H's article on the fatal effects of chloroform. After alluding to the different opinions that have been entertained in reference to the cause of the bad effects of chloroform, and detailing some experiments, he says:

The Fusel oil of the apothecaries, treated with water and bleaching salt in all respects as if it were alcohol, with a view to the production of chloroform, gave a product nearly like pure Fusel oil in smell, and having a specific gravity in these several preparations of—

0,8236
0,8225
0,8224

While that of pure Fusel oil is, 0,8124. It gave of chlorine, 1,35 per cent when evaporated to dryness with water-lime, the chlorine in which had been previously determined. This was undoubtedly due to the chloroform derived from the trace of alcohol still present in the Fusel oil.

That such might have been expected as the result, will be evident when it is considered that Fusel oil is but slightly solnble in water, and would of course, from the ouset, float on the surface of the mixture.

Experiments made with alcohol to which was added impure methyl alcohol (wood spirit) gave good chloroform.

Experiments with the product of distillation resulting from the mixture of pure Fusel oil, water and bleaching salt, upon man and inferior animals, were made under quite varied circumstances.

A practising physician accustomed to the administration of chloroform, inhaled the vapor of this product for fourteen minutes, without any marked anæsthetic effect, or any other effect than slight irritation of the bronchial tubes.

Two rats, one full grown, were successively subjected to the action of this agent, poured upon cotton to facilitate evaporation, the tuft of cotton and the animal being placed on the bottom of a covered becker glass. The air was renewed from time to time with the aid of a bellows. At the end of an hour no aneæsthetic effect had been produced upon the full grown rat, and at the end of forty minutes none on the smaller animal.—They were then exposed to the action of the vapor of chloroform, and in less than two minutes were insensible.

The experiment was repeated with kittens about a week old, with like results, except that they were longer in becoming insen sible.

On a subsequent day two kittens were exposed to the vapor of the above body, each under a separate bell glass, while two others, in all respects similarly situated, except that the latter breathed merely confined air, for one hour, without its being apparent that the vapor had produced any deleterious effect. When taken out, all appeared quite alike so far as activity was concerned, or disposition to seek legitimate nourishment.

At a period several weeks later, the experiments were repeated with the same kittens, in the use of a fresh preparation of the above body, repeatedly distilled from chloride of calcium. There was in the course of an hour, an appreciable lethargic effect which was not so marked where confined air was alone inhaled, but in no instance attained such a degree that upon the removal of the bell glasses, the animals did not at once resume unimpaired, the possession of all its powers.

These experiments led to the conviction that Fusel oil, when treated as in the manufacture of Chloroform, substituting Fusel oil, is not changed, and of course, that the Fusel oil present in alcohol in the ordinary manufacture of chloroform does not yield a poison, which taken with the chloroform has produced the fatal effects.

While the Fusel oil vapor and the impure chloroform which Gregory had recognized, could be inhaled without difficulty, the article received from Dr. Currie, by its violent irritation, closed the glottis almost instantly.

Upon the presumption that the bad attribute was due to the mode of purification, a quantity of good chloroform was repeatedly distilled from concentrated sulphuric acid, and another from chloride of calcium. The products in both cases were perfectly good, and are so still, now nine months from the date of distillation.

Having tried alcohol of various degrees of purity and strength, and having subjected good chloroform repeatedly to different methods of purification, it remained to try different samples of bleaching salt with varying proportions of alcohol. Accordingly several varieties in the market were procured, and a series of experiments undertaken by Mr. Gould, of the Laboratory of the Lawrence Scientific School.

The combinatians of alcohol, bleaching salt, water, temperature, time and mode of distillation, made to meet the inquiry required fifty successive preparations of chloroform, the detailed results of thirty-one of which will appear in the official proceedings of the Association.

From these and the foregoing series of experiments, it appears—

1st. That good chloroform does not spontaneously change in a period of nine months, and perhaps longer.

2d. That the bad chloroform, containing free chlorine and hydrochloric acid, may be produced by using a bleaching salt of great strength with a quantity of alcohol disproportionately small.

3d. That the bad chloroform may be produced by receiving the distillate into water, so as immediately to withdraw the alcohol from the chloroform.

4th. That the bad chloroform may be produced by passing chorine directly into chloroform.

5th. That no formula for its manufacture can be relied upon as a guide, since bleaching salts vary in strength when derived from different factories, and vary with age. In the foregoing experiments the range is from 15 to 30 per cent.

6th. That quick lime added to the mixture does not promote the economy of manufacture.

7th. That the chlorine and hydrochloric acid of bad chloroform, as observed by Dr. Dwight, may be removed by agitation with a little alcohol.

8th. That the ill effects observed in the administration of chloroform are not due to the presence of chlorine as the irritation is such when it is attempted to inhale it, as to prevent inhalation altogether.

9th. That the ill effects are not due to any poisonous product arising from the action of bleaching salt on the small quantity of Fusel oil, in the alcohol employed in the manufacture of chloroform.

10th. That the ill effects are due to peculiarities of constitution or temparament of some patients, and in a few rare cases to want of attention or judgment on the part of the person administering it.

